# Positive and Negative Feedbacks and Free-Scale Pattern Distribution in Rural-Population Dynamics

**DOI:** 10.1371/journal.pone.0114561

**Published:** 2014-12-04

**Authors:** Concepción L. Alados, Paz Errea, Maite Gartzia, Hugo Saiz, Juan Escós

**Affiliations:** 1 Pyrenean Institute of Ecology (CSIC), Zaragoza, Spain; 2 Pyrenean Institute of Ecology (CSIC), Nuestra Señora de la Victoria s/n, Jaca, Huesca, Spain; 3 Department of Animal Production and Food Technology, Huesca Polytechnic School, Zaragoza University, Zaragoza, Spain; University of Zurich, Switzerland

## Abstract

Depopulation of rural areas is a widespread phenomenon that has occurred in most industrialized countries, and has contributed significantly to a reduction in the productivity of agro-ecological resources. In this study, we identified the main trends in the dynamics of rural populations in the Central Pyrenees in the 20^th^ C and early 21^st^ C, and used density independent and density dependent models and identified the main factors that have influenced the dynamics. In addition, we investigated the change in the power law distribution of population size in those periods. Populations exhibited density-dependent positive feedback between 1960 and 2010, and a long-term positive correlation between agricultural activity and population size, which has resulted in a free-scale population distribution that has been disrupted by the collapse of the traditional agricultural society and by emigration to the industrialized cities. We concluded that complex socio-ecological systems that have strong feedback mechanisms can contribute to disruptive population collapses, which can be identified by changes in the pattern of population distribution.

## Introduction

The depopulation of rural areas, which is common in most developed countries, has, paradoxically, increased in parallel with global population growth [1], which is one of the most important challenges facing civilization [2]. At a time when many areas are faced with problems associated with rapid human population growth, others are confronted with the effects of the rapid population loss. The effects of those changes on the distribution of populations have important implications for landscape conservation and socioeconomic life in rural areas because of the high social costs associated with low population densities. In addition, steady increases in the global population and the global food-crisis of 2007–2008, which resulted in almost a doubling of international wheat and maize prices within two years [3], have brought greater attention to the need to conserve agro-ecological-based production systems to enhance food security and conserve agro-biodiversity, soil, and water [4].

The dynamics of human population growth are complex, with multiple processes occurring sequentially or simultaneously [2], [5]. Many studies have investigated the multitude of factors that influence population growth as a basis for making predictions about the future [6]–[9]. In general, human population dynamics are characterized by steady increases that are occasionally interrupted by collapses that are caused by density-dependent and density-independent factors, which are followed by rapid increases [10]–[12]. For thousands of years, human populations grew very slowly, depended on natural resources and adapted to the changes in their environment [5], [10], [13], [14]. Two hundred and fifty years ago, the exploitation of fossil fuels during the Industrial Revolution allowed the global population to increase from about 500 million to 6 billion in 2000 [2], [10]. The most significant changes in the size and distribution of human populations occurred in the 1960s, when per capita growth rate was at the maximum. Thereafter, populations decreased steadily in rural areas [2], [15].

Rural alpine societies are complex systems that were based on the exploitation of natural resources, where livestock grazing and crops have led to a distinctive complex anthropogenic landscape in which the close connections between land-use, livelihood, and socio-cultural factors influence the landscape [16], [17]. Those complex systems organize at multiple scales through the flux of resources and information [18], [19]. As those complex systems develop, the bonds among their components, information, entropy, order, and structure increase, which optimizes the distribution of energy and matter among group members [20], [21]. In time, those systems develop into self-organized critical structures or states [22]. For instance, in ancient human hunter-gatherer societies, the effective resource supply increases non-linearly to the power of population size, which indicates that densely distributed populations made the most efficient use of resources from the environment [21]. Other mammalian social systems [23] exhibit scaling laws that are similar to the scaling law that occurs in humans [18], [19]. Thus, like many other complex systems, human societies self-organize and generate self-similar patterns that are free of scale [19]; i.e., the response to what happens at the smallest scale in a dynamic process is not restricted to this scale; rather, it can influence the entire hierarchical structure. Those complex systems evolve and organize over time and are resilient to perturbations unless certain thresholds are crossed. Once those thresholds are exceeded, a transition to a different structure and process begins, which has had important implications for humans. Simple power laws of either emergent patterns or fluctuations can describe the statistical properties of the system because the distribution of cluster sizes leads to a distribution of fluctuation lifetimes; local perturbations propagate over all length scales, and the distribution of lifetimes can be calculated from the distribution of cluster size [22].

In mountainous areas of Europe, human emigration from rural areas to industrialized cities and the mechanization of agriculture, which reduced the demand for labor, lead to substantial depopulation [24]–[26] and, subsequently, the loss of agro-ecological lands caused by woody encroachment on abandoned lands [27]–[29]. After the 1960s, rural areas in the Pyrenees experienced significant depopulation [26], [30] while the population of the nearest industrialized city, Zaragoza, doubled (Table S1), which contributed to the woody encroachment of rural agricultural and grasslands areas [31]–[34]. For many years, much attention has been paid to the growth of global and metropolitan populations and the question of whether the global population will increase until the resources are exhausted and the population crashes [10] or, whether limited resources will limit the population growth rate until a more-or-less equilibrium is reached [35]. Given the current rate (225,000 persons/day) at which the human population is increasing [36], it is evident that additional grasslands will be needed to fulfill human food requirements. Properly managed grasslands can provide food security and alleviate poverty for millions of individuals. Yet, changes in climate and land use are driving those ecosystems toward irreversible degradation and reduced productivity [17], [37], [38].

**Figure 1:**
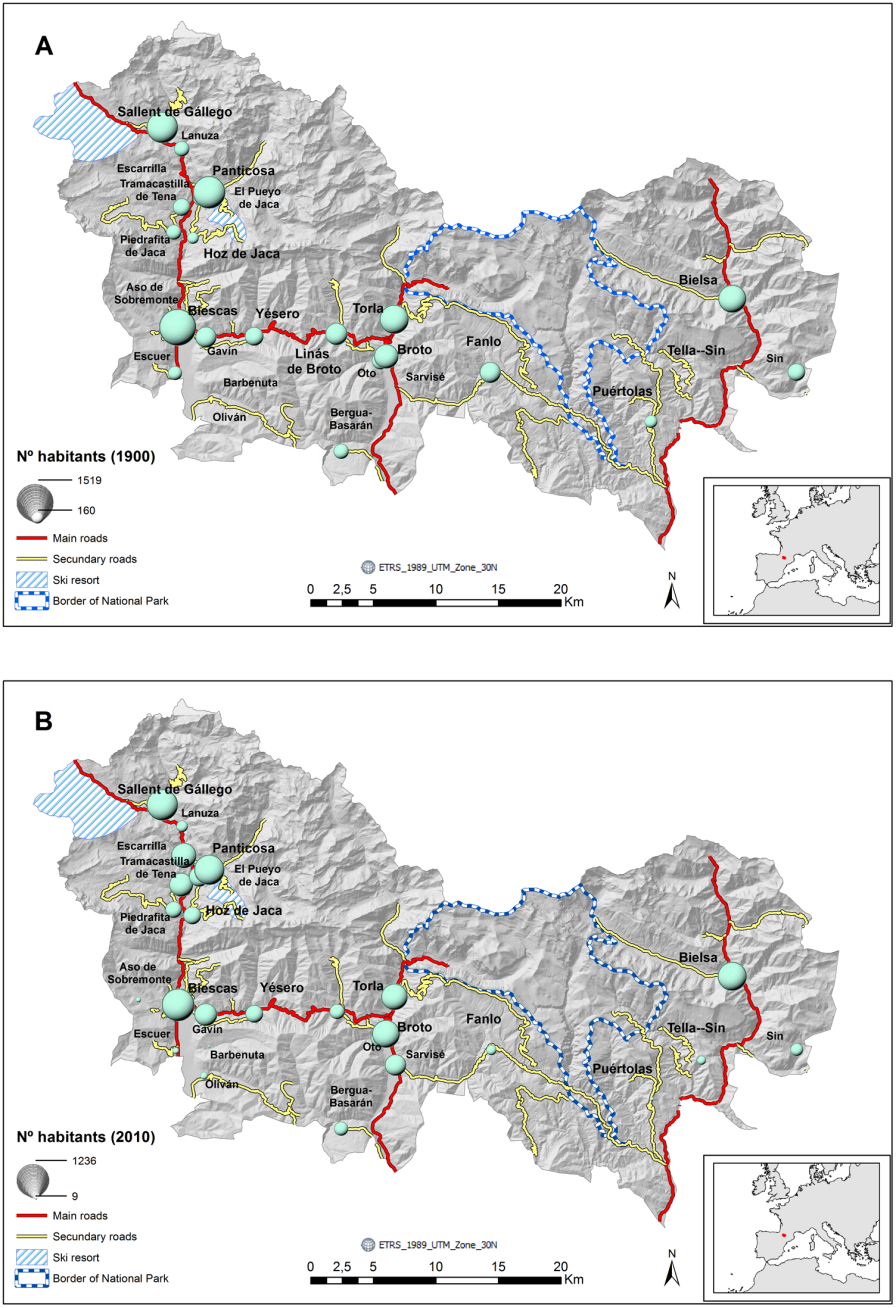
Study area in Spanish Central Pyrenees, which included 26 municipalities, location of Ordesa and Monte Perdido National Park, ski resorts, and main and secondary roads. Dot sizes were scaled to the size of the population in each municipality for the year (A) 1900 and (B) 2010.

In this study, the objectives were to identify the main trends in rural population dynamics and to confirm whether density-dependent positive feedback has contributed to the resilience of the rural populations of Spanish Central Pyrenees (Fig. 1). A positive feedback can occur between per capita growth rate and population size [2], which indicates that cooperative relationships and aggregation can enhance the growth of human populations [39], as it can in animal populations [40] and can increase population carrying capacity [39]. Complex socio-ecological systems that have strong feedback mechanisms can exhibit emergent patterns that reflect the level of system self-organization [19], which can be used as an early indicator of socio-ecological stability (resilience). Although emergent spatial patterns as an indicator of critical transition have been the subject of considerable study in the last decade [41]–[45], the formation of patterns in socio-ecological societies has received limited attention [18], [19], [21], [46].

In this study, we identified the best-fit model of rural populations based on density-independent and density-dependent estimations, and assessed the importance of positive and negative feedbacks in rural populations. We hypothesized that population regulation negative feedback occurred in the first half of the 20^th^ C, when rural population were dependent on natural resources, and density-dependent positive feedback occurred in the second half of the 20^th^ C and early 21^th^ C, when the increase in human population size promoted economic and social development in modern societies. In addition, we assessed whether the population distribution formed self-similar structures and, if so, how these scaling properties differ among socio-economic conditions. We hypothesized that the rural population size exhibited a free-scale distribution (power law scaling) that, when disrupted, became randomly distributed (exponential distribution), which would be indicated by the change in the value of the power law exponent.

## Materials and Methods

### Study Area

The study area was a 138.4-km^2^ portion of the Spanish Central Pyrenees (42° 36′ N, 0° 00′E), where the elevation ranged between 600 and 3340 m (Fig. 1). Historically, the local economy was based on traditional agriculture, mostly sheep and cattle production, in which alpine and subalpine pastures were used for grazing in summer [47]. Between 1965 and 1976, five alpine ski resorts and associated tourist infrastructures were built in the Spanish Central Pyrenees, which led to significant changes in the population and in pastoral activities in the area [48], [49]. Two of those resorts were within our study area (Fig. 1). The study included 26 municipalities, some of which were amalgamated in the 1960s, which led to the formation of 11 municipalities in the 1970s. The study area had one of the lowest population densities in Spain (4.45 hab. km^−2^), which was much lower than the density of the Autonomous Community of Aragón (28.20 hab. km^−2^), of which it is a part. Table S1 provides number of inhabitants in the 26 municipalities in the Spanish Central Pyrenees since 1900 to 2010.

### Data collection and analysis

The Instituto Nacional de Estadística [50] and the Instituto Aragonés de Estadística [51] provided the data that were used to quantify the size and structure of the human populations. The Archivo Histórico Provincial de Huesca and the Delegación Provincial de Huesca provided the livestock data for the period 1900–2010, which were assessed at 10-yr intervals. The livestock data from the 26 municipalities between 1965 and 2010 were pooled into 11 post-1960s municipalities. To calculate the number of small-livestock units in the area, we assumed that six sheep are equivalent to one cow [52]. Employment data in the main economic sectors: primary sector (e.g., farming) and tertiary sector (services), which were used to characterize the economic structure in Spain, were obtained from the Instituto Nacional de Estadística. Population data were available for the 26 municipalities for the period covered by this study; however, both livestock and employment data were assessed based on the 11 municipalities created in recent decades.

### Calculation of the population parameters

The mean (µ) and the variance (σ^2^) of the log population growth rate describe the normal probability of the distribution of the future log population size, assuming density independence *N_t+1_* = *N_t_ exp* (*µt*); where *N_t_* is population at time *t*, and *N_t+1_* is population at time *t+*1. When *µ*>0, the population increases, and, when *µ*<0, the population decreases. The parameter values were derived from the parameter estimates and the variance from the residuals of the linear regressions of the log population growth rate over a time interval against the time interval. Variance of log population growth rate increases with the time; therefore, to meet the assumption of equal variance, the rate of population’s change and the time elapsed were transformed by dividing both by (*t_i+1_*–*t_i_*)^0.5^ [53]. Thus we regressed *log* (*N_i+1_*/*N_i_*)/(*t_i+1_*−*t_i_*)^0.5^ against (*t_i+1_*−*t_i_*)/(*t_i+1_*−*t_i_*)^0.5^ where *N_i_* is population at time *t_i_* and *N_i+1_* is population at time *t_i+1._* This transformation makes the variance in the transformed population to change equal to the variance for any time interval *σ* ^2^.

In addition, population counts were fit to a logistic population dynamics model (Ricker model), which assumes that population growth is density dependent. The Ricker model is expressed as: *N_t+1_* = *N_t_ exp* [*µ*(1−(*N_t_*/*K*)] [54].

We fit the models to the data using nonlinear least squares regression of *log* (*N_i+1_*/*N_i_*)/(*t_i+1_*−*t_i_*)^0.5^ against [(*t_i+1_*–*t_i_*)/(*t_i+1_*–*t_i_*)^0.5^] (1−*N_i_*/*K*). We used the corrected Akaike's Information Criterion, AIC, [55] to select the best-fit model.

### Statistical analysis

To identify the main factors that have influenced the dynamics of the human population in the study area in the last century, we used generalized linear mixed-effects models (GLMMs) that were fitted based on restricted maximum likelihood (RMLE). We used the *lme* function in the *nlme* library of the R package [56], and the analyses followed the protocol of Zuur [57]. The optimal structure of the random component was identified based on the lowest Akaike’s Information Criterion (AIC). Analyses of the distribution of the residuals and a q-q plot tested the validity of the model.

We assessed the effects of livestock density, agricultural area (ha), and distance to the nearest county capital on the changes in the density of the human populations within each municipality. Location (municipality) and year can influence the observed differences in population densities; therefore, we evaluated the random effects of “municipality” and “year” on the intercept of the model.

The relationship between the carrying capacity and the total land allocated to croplands and grasslands (ha) where calculated by GLMM with municipality as random effect factor to control the variation due to municipality.

To assess the importance of density dependent positive and negative feedback in the rural populations in the two distinctive periods in the 20^th^ C (the first half dominated by agronomic economy, and the second half dominated by the service sector) we regressed per capita growth rate against the log of population size in the period 1900–1950 and in the period 1960–2010.

We calculated the probability cumulative distribution function (CDF) for each population census (S) between 1900 and 2010 for the 26 municipalities in the study area. To construct the CDF (*S*), the *n* observed values (*s_i_*) were ranked from lowest to highest (*i* = 1… *n*). The probability of finding an observation less than or equal to *s_i_* in the CDF follows a power law: P (*S*≤*s_i_*) = *k s*_i_ *^Υ^* ^+1^ [58]. The exponent *Υ* reflects the heterogeneity in population sizes: the larger the absolute value of *Υ*, the more heterogeneous is the population size. To estimate the exponent, we used maximum likelihood because it is the best mathematical approach [58]. The goodness-of-fit of the power law distribution was assessed based on the coefficient of determination R^2^.

## Results

Akaike’s Information Criterion (AIC) indicated that the density-independent model had the best fit to the data of the dynamics of rural populations in the Spanish Central Pyrenees in the 20^th^ C and early 21^st^ C with the exception of Panticosa population that fitted better to the Ricker model (Table 1). All of the populations except those in Panticosa, Escarrilla, and Sallént de Gállego had negative growth rates between 1900 and 2010. Nevertheless in rural economies, we expected the allocation of land to agriculture and pastures have influenced carrying capacity (maximum population size). We estimated the maximum carrying capacity of each municipality based on the density-dependent model, and assumed that each population was dependent on internal resources.

**Table 1:** Parameter estimates for density independent (per capita growth rate, µ±se) and density dependent (per capita growth rate, *µ* and carrying capacity *K*) population models, residual variance, σ^2^, and Akaike’s Information Criterion (AIC) of mountain rural populations in the Spanish Central Pyrenees.

Municipality	*σ^2^*	*µ*	se	AICc	*µ*	*K*	*σ^2^*	AICc
Aso de Sobremonte	0.0805	−0.0307	0.0270	9.154	−0.0375	1142.35	0.0802	11.732
Barbenuta	0.0031	−0.0109	0.0053	−30.805	−0.0142	953.48	0.0031	−27.202
Biescas	0.0030	−0.0019	0.0052	−31.469	0.0247	1119	0.0026	−29.509
Escuer	0.0037	−0.0140	0.0058	−28.871	−0.0263	287.12	0.0034	−26.134
Gavín	0.0029	−0.0103	0.0052	−31.677	−0.0103	568700000	0.0029	−28.015
Oliván	0.0036	−0.0136	0.0057	−29.080	−0.0169	1378.68	0.0036	−25.511
Piedrafita de Jaca	0.0170	−0.0153	0.0124	−10.544	−0.0153	143700000	0.0170	−6.884
Hoz de Jaca	0.0005	−0.0074	0.0021	−53.100	−0.0154	240.35	0.0004	−50.370
El Pueyo de Jaca	0.0049	−0.0068	0.0067	−25.475	0.0071	68.54	0.0047	−22.368
Panticosa	0.0205	0.0004	0.0041	−8.311	0.0240	630.01	0.0017	−34.657
Escarrilla	0.0025	0.0012	0.0047	−33.713	0.0172	189.33	0.0024	−30.427
Lanuza	0.4485	−0.0143	0.0639	28.721	0.0894	114.32	0.4100	31.311
Sallent de Gállego	0.0065	0.0039	0.0077	−22.075	0.1080	716.47	0.0043	−23.491
Tramacastilla de Tena	0.0016	−0.0035	0.0038	−39.080	0.0125	163.18	0.0014	−36.649
Yésero	0.0078	−0.0127	0.0084	−19.916	−0.0127	76960000	0.0078	−16.250
Bielsa	0.0023	−0.0057	0.0046	−34.663	−0.0057	573100000	0.0023	−30.998
Bergua−Basarán	0.1402	−0.0234	0.0357	14.771	−0.0234	325500000	0.1403	18.440
Broto	0.0018	−0.0032	0.0040	−37.815	0.0177	263.57	0.0016	−35.272
Oto	0.0017	−0.0124	0.0039	−38.519	−0.0199	558.68	0.0015	−35.623
Sarvisé	0.0033	−0.0094	0.0054	−30.381	−0.0096	16720	0.0033	−26.717
Fanlo	0.0322	−0.0182	0.0171	−2.897	−0.0182	579900000	0.0322	0.744
Puértolas	0.0069	−0.0126	0.0079	−21.444	−0.0126	370900000	0.0068	−17.785
Sin−Salinas	0.0047	−0.0125	0.0065	−26.038	−0.0159	1174.37	0.0065	−18.430
Tella	0.0038	−0.0036	0.0059	−28.472	0.0061	217.60	0.0058	−19.712
Linás de Broto	0.0040	−0.0169	0.0060	−27.884	−0.0285	637.81	0.0036	−25.361
Torla	0.0012	−0.0071	0.0033	−42.232	−0.0071	562100000	0.0012	−38.568

In the Spanish Central Pyrenees, the carrying capacity, derived from the Ricker model and the amount of land allocated to croplands and grassland (ha) derived from 1980s Landsat images [34], were significantly positively correlated (F_1,24_ = 13.88, P<0.001) after controlling the effect of municipality, introduced as a random effect factor in the GLMM. Thus, as expected, population density throughout the 20^th^ C was positively correlated with livestock density (slope = 0.36±0.07, F_1,120_ = 23.92, P<0.001) and amount of agricultural area (slope = 0.008±0.002, F_1,8_ = 12.00, P<0.01), and negatively correlated with distance to the county capital (slope = −0.14±0.07, F_1,8_ = 3.90, P = 0.08).

**Figure 2:**
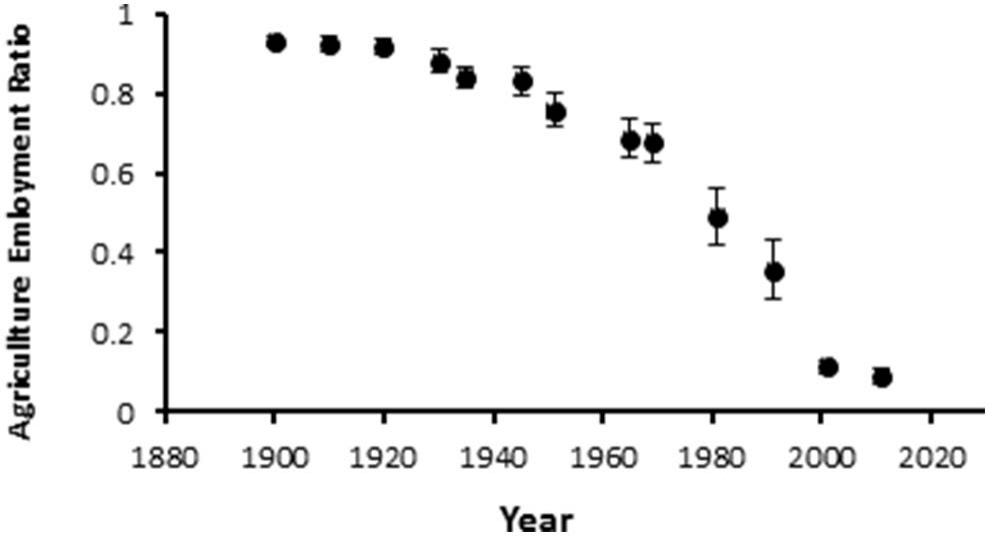
Employment rate in the agricultural sector in the 20^th^ C and early 21^st^ C in the Spanish Central Pyrenees.

Livestock and agriculture production are the main activities in the area, which involved >90% of the active population in 1900, 70% in the 1970s, and 1% in 2010 (Fig. 2). The industry sector was not present in the study area, and the service sector has provided a source of employment for those not working in the agricultural sector. Thus, the importance of density dependence feedback in the rural populations was expected to vary in the periods 1900–1950 and 1960–2010.

**Figure 3:**
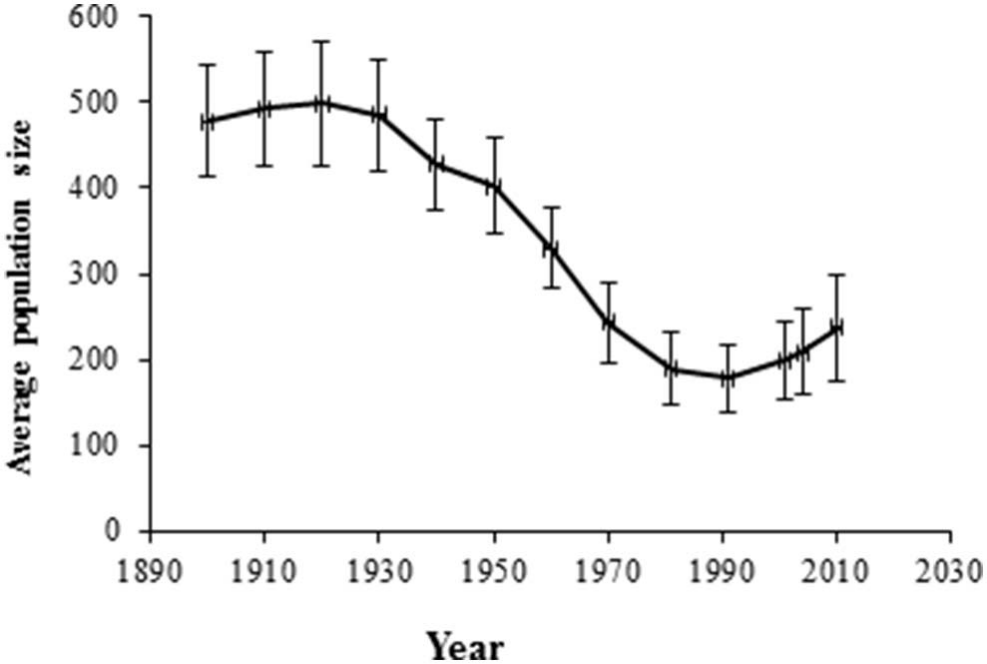
Averaged human population sizes in 26 municipalities in the Spanish Central Pyrenees, between 1900 and 2010. Error bars indicate the standard error.

**Figure 4:**
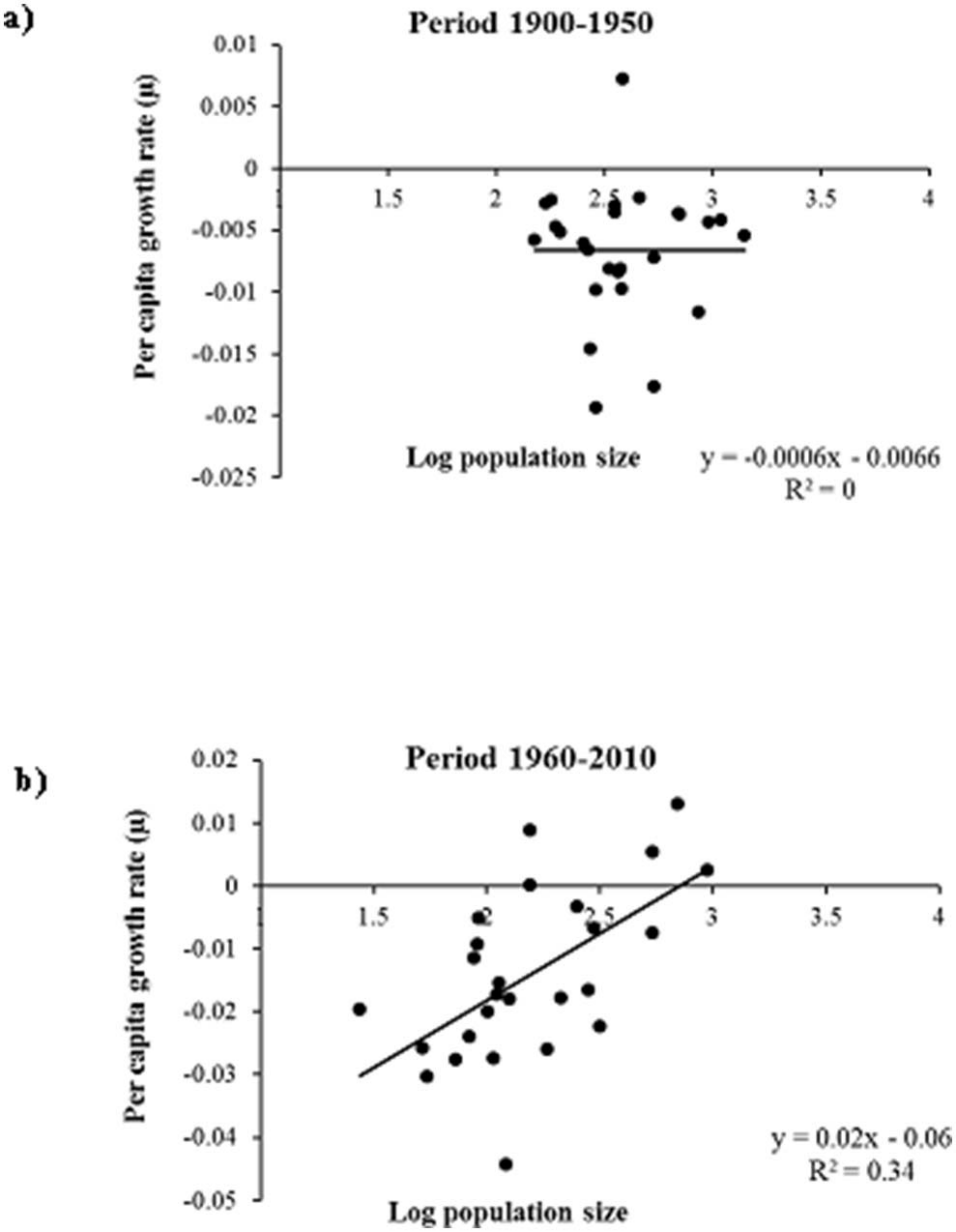
Per capita growth rate and log of population size for the periods (a) 1900–1950 and (b) 1960–2010 in the Spanish Central Pyrenees.

Before 1950, the rural populations were stable; however, after 1950, the human population decreased significantly (Fig. 3), t = −2.15, P<0.0337 (F_11,110_ = 29.12, P<0.0001). A positive correlation between per capita growth rate and log population size indicates a positive feedback, and a negative correlation indicates a negative feedback. Between 1960 and 2010, there was a positive feedback; but, in the first half of the 20^th^ C, the per capita growth rate was stable, which reflected the negative feedback effect in the largest population in the area (Fig. 4).

**Figure 5:**
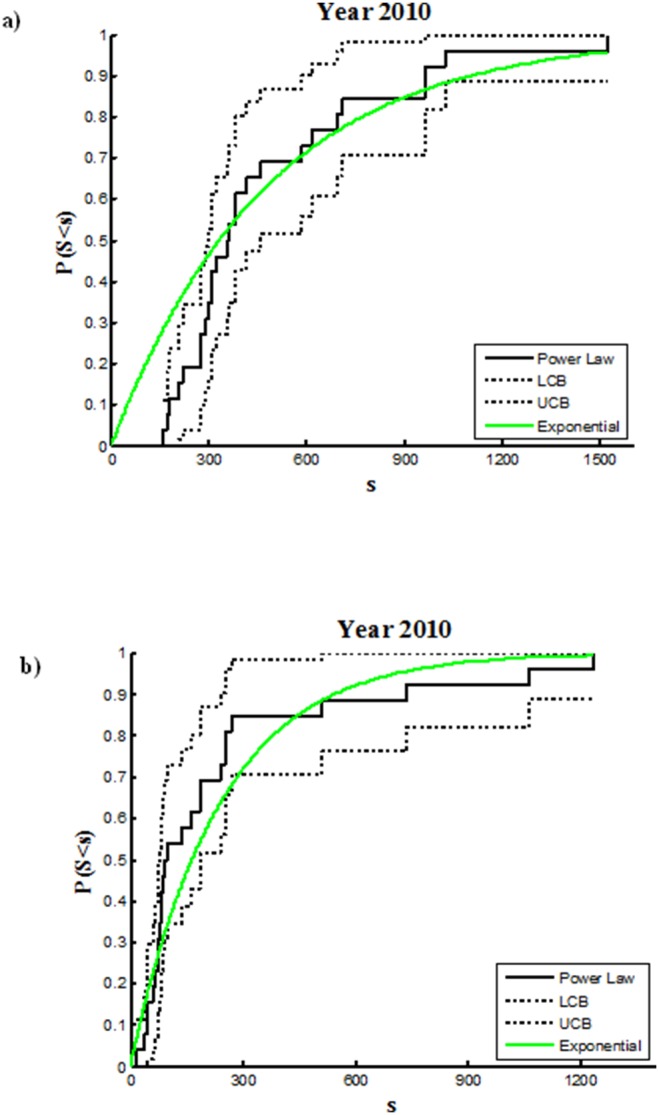
Empirical cumulative distribution function and confidence bounds that describe the probability that a random population (S) is equal to or lower than (s). In green the theoretical exponential function: (a) year 1900; (b) year 2010. The power-law coefficient of determination is 0.842 and 0.560 for the year 1900 and 2010, respectively.

The distribution of the populations changed over time: some of the population nuclei disappeared (but not the municipalities where they were administratively included) or were much reduced, but others persisted or increased in size. Analyses of the power law distribution of the population sizes of the 26 municipalities at each 10-yr census interval revealed that the power law best fit the population size distribution in the first half of the 20^th^ C, which indicated that population sizes had a wide range of size scales, with many small populations and relatively few large ones. The values of the exponent (*Υ*) were similar throughout the first half of the 20^th^ C, and fit well the power law distribution, as indicated by the coefficient of determination, R^2^ (Fig. 5a, Table 2). The power-law function was not a good fit in the last half of the 20^th^ C (Fig. 5b, Table 2), and the exponent of the power law function decreased dramatically, which indicated a randomization of the population size distribution. The maximum likelihood estimation (*mle*) indicated the poor fit of the power law distribution in that period (Table 2).

**Table 2:** Power low parameters, maximum likelihood estimator, *mle* and determination coefficient, R^2^, of the human population distribution between 1900 and 2010 in 26 municipalities in the Spanish Central Pyrenees.

Human population													
Year	1900	1910	1920	1930	1940	1950	1960	1970	1981	1991	2001	2004	2010
Exponent (*Υ*)	0.3764	0.3531	0.2961	0.3393	0.4007	0.2460	0.2484	−0.1885	−0.3835	−0.3434	−0.1970	−0.2257	−0.2330
intercept	−9.1287	−0.9013	−8.6461	−8.9024	−9.1349	−8.0900	−7.8464	−4.9755	−3.7646	−3.9365	−4.9970	−4.5968	−4.6271
*mle*	−0.2535	−0.2632	−0.2931	−0.2216	−0.1049	−0.2316	−0.2890	−0.4543	−0.5300	−0.5124	−0.4820	−0.5233	−0.5552
R^2^	0.842	0.847	0.8067	0.7867	0.8167	0.831	0.819	0.704	0.745	0.729	0.662	0.611	0.560

All distributions fitted with *P*-values<0.001.

**Figure 6:**
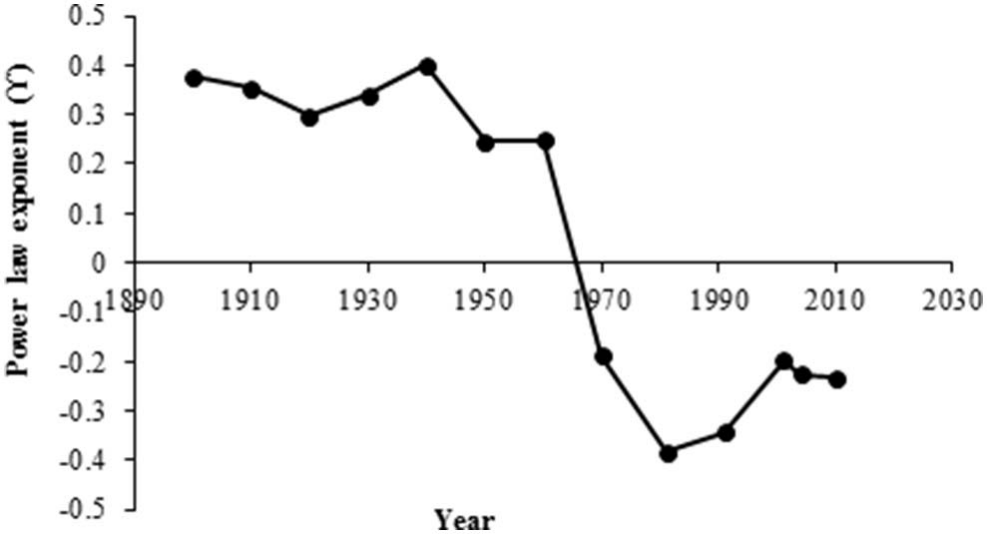
Power law exponent of the human population distribution between 1900 and 2010 in 26 municipalities in the Spanish Central Pyrenees.

The slope of the power law and the heterogeneity of the population size distributions were positively correlated; i.e., the higher the slope, the more heterogeneous were the population sizes and the greater were the differences between large and small towns. Thus, the change in the power-law exponent in the 20^th^ C reflects the change in the spatial distribution of the population. Between 1900 and 1960, the *Υ* parameter was stable at about 0.4 (Fig. 6). After 1970, the parameter *Υ* decreased dramatically to values close to −0.4, before it began to increase in the last decade.

## Discussion

In the Spanish Central Pyrenees, rural populations fit well to the density-independent model for the entire period evaluated in our study; however, in the first half of the 20^th^ C, rural society was characterized by competition among individuals for limited resources (negative feedback), which is common in rural populations that are dependent on natural resources. At the global scale, studies have shown that negative feedback processes occur in human populations [2], [59], [60], and socio-economic and natural resources are the forces that drive that dynamic [2].

In the second half of the 20^th^ C., the rural populations exhibited a positive feedback between per capita growth rate and log population size, which occurred in the global population between 1700 and 1960 [2]. Those results are consistent with the hypothesis that cooperative human interactions exert positive feedback on population growth [39], [61], [62]. Those complex socio-ecological systems that have strong positive feedback mechanisms exhibited emergent free-scale population distribution patterns that reflected the level of system self-organization.

To model population growth, ecologists and population biologists have used the density-dependent model of population dynamics [63], which is based on the assumption that, in the presence of unlimited resources, i.e., space and food, populations will grow exponentially. If resources become limited, however, the growth rate decreases until the population is below the maximum population size that the resources can support.

Density-independent and density-dependent models indicated that, between 1900 and 2010, most of the rural populations in the Central Pyrenees had negative per capita growth rates, with the exception of populations that were close to ski resorts, e.g., Sallent de Gállego and Panticosa. Rural populations are likely to be dependent on natural resources and, consequently, to be density-dependent; however, when they are well below the carrying capacity, they fit well density-independent models. Although, as in the pre-industrial era [12], [13], in model rural economies, the allocation of land to agriculture and pastures is likely to influence carrying capacity.

A strong positive correlation between carrying capacity and the amount of land allocated to agriculture and livestock production reflected the dependence of the rural populations on agricultural resources. Studies have demonstrated the need to have data on food supply to adequately fit the logistic model of human population dynamics, which suggests that increases in human populations are a function of an increase in food availability, which occurs in non-human populations [35]. In our study, the GLMM indicated a positive correlation between ecosystem services such as cultivable land and livestock density, and the negative effect of distance to developing socio-economic centers (county capital) on population dynamics, which confirm that natural resources have significant effects on population density.

Various mechanisms such as reduced probability of finding mate, impaired group dynamics, or conditioning of the environment can cause density-dependent positive feedback, depensation [64], which occurs in many species of animals and plants, e.g., predator detection increases with group size in Spanish ibex [65], hunting success increases with population size in social hunters as *Lycaon pictus* [66] or seed density is lower in small populations because they likely attract less pollinators [67]. Density-dependent positive feedback mechanisms in human populations are the basis for the hypothesis that aggregation and cooperation in human societies accentuate the growth of populations [2], [39].

The positive correlation between per capita growth rate and population size in the Spanish Central Pyrenees in the second half of the 20^th^ C was consistent with the hypothesis that human population size promotes economic development and enhances technological innovation and food production [62], [68], which suggests that high population densities in rural areas increase living standards. That positive feedback effect however, reaches an upper limit as population density increases to a point at which the resources are insufficient to meet human needs; hence, the reduction in per capita population growth as population density increases. Cooperative feedback can occur at low population densities [39] but, at high densities, competition and negative feedback dominate the dynamics [2], [61].

Other mechanisms external to the populations can contribute to the increase in the population, independent of the population size: e.g., subsides given to rural populations to help maintain the population size. In Spain, however, rural populations continue to decline despite subsides received since the country joined the European Community.

Resource availability, socio-economic crises, and climate change have created delayed feedback loops and long-term cycling oscillations [14], [61], [69], which reflects the difficulty in preventing population collapses. Although the reduced per capita growth rate in the Spanish Central Pyrenees has been reversed in some of the municipalities because of the development associated with ski resorts [70], the reduced population size will affect population dynamics for several decades because there are still numerous small villages in the hands of elderly farmers, who will retire soon. In the near future, most of the farms will be removed from agriculture and livestock production, which will favor the transition of pastures and cultivated areas to woodlands after abandonment, exacerbating the loss of productive pastures and crops caused by woody encroachment [34].

Population reduction is the most direct measure of depopulation in rural areas; however, complex socio-ecological systems that have strong feedback mechanisms can respond non-linearly, which can lead to catastrophic population collapses. In the Spanish Central Pyrenees, the rural mountain society exhibited a fractal-like structure in which population size increased free-scale on multiple scales. Self-similar group size distribution free of scale occurs in some hunter-gather societies [18], [19] and other mammal societies [23]. The mechanisms underlying the distribution patterns and the buffering capacity of the complex system dictate the change in distribution patterns. In our study, the scaling exponent *Υ* of the power law distribution reflected the capacity of the system to resist perturbation. Even under the extreme conditions of the Spanish Civil War, the distribution patterns of the rural population persisted in spite of the reduction in the population. After the industrial development in the 1960s, however, the population distribution patterns changed dramatically, which reflected the important changes that had occurred in the area. Although the power-law distribution has been identified in some biological systems [71], [72], to our knowledge, temporal changes in population distribution power laws have not been investigated. Our results are consistent with the hypothesis that a change in the scaling exponent provides information about the level of system self-organization and complexity [73], [74]. With the population declining, the free-scale population size distribution was lost, which resulted in a homogeneous population distribution. The long-term positive correlations between agricultural activity and population size generated a scale-free population distribution. When population sizes were reduced because of the breakdown in the traditional agronomic society and the emigration of individuals to the industrialized cities, the scale-free distribution was disrupted. In the same period, the population of the industrialized city of Zaragoza increased from 99,118 inhabitants in 1900 to 674,725 in 2010, while the population in the study area decreased from 12,424 to 6,163 in the same period (SI).

## Conclusions

In the Spanish Central Pyrenees, two limiting factors regulated rural populations: (*i*) negative feedback occurred when limited resources did not provide sufficient work and benefits to the population, and (*ii*) positive feedback occurred at low population densities, when a minimum population size was required to avoid the effects of stochasticity and to ensure that the basic services needed to maintain the rural populations were present. Changes in the structure of a population distribution can provide important clues about the likelihood of population collapses, and is more informative and cost-effective measurement than are mean rates in assessing the relative risks of population extinction and as an aid in establishing the priority of rural developments projects.

## Supporting Information

Table S1 **Number of inhabitants in the 26 municipalities in the Spanish Central Pyrenees since 1900 to 2010.**
(DOCX)
