# Supplementary material for: Positive and Negative Feedbacks and Free-Scale Pattern Distribution in Rural-Population Dynamics
Source: PLoS One. 2014 Dec 4;9(12):e114561. doi: 10.1371/journal.pone.0114561 (PMC4256442; doi:10.1371/journal.pone.0114561)
Supplement: Table S1 — Number of inhabitants in the 26 municipalities in the Spanish Central Pyrenees since 1900 to 2010. (DOCX) [file pone.0114561.s001.docx]

| Supplementary information (SI). Number of inhabitants in the 26 municipalities in the Spanish Central Pyrenees since 1900 to 2010 | | | | | | | | | | | | |
| --- | --- | --- | --- | --- | --- | --- | --- | --- | --- | --- | --- | --- |
| **Municipality** |  |  |  |  |  | **Year** |  |  |  |  |  |  |
|  | 1900 | 1910 | 1920 | 1930 | 1940 | 1950 | 1960 | 1970 | 1981 | 1991 | 2001 | 2010 |
| Aso de Sobremonte | 296 | 314 | 285 | 292 | 289 | 257 | 239 | 152 | 94 | 89 | 80 | 89 |
| Barbenuta | 380 | 402 | 405 | 366 | 336 | 242 | 119 | 14 | 6 | 2 | 10 | 13 |
| Biescas | 1519 | 1583 | 1656 | 1455 | 1034 | 1211 | 1093 | 1010 | 911 | 817 | 885 | 1236 |
| Escuer | 177 | 193 | 162 | 199 | 174 | 169 | 152 | 91 | 60 | 55 | 43 | 38 |
| Gavín | 308 | 325 | 296 | 278 | 250 | 173 | 128 | 110 | 85 | 78 | 78 | 99 |
| Oliván | 414 | 402 | 455 | 378 | 341 | 294 | 230 | 151 | 98 | 98 | 83 | 93 |
| Piedrafita de Jaca | 356 | 359 | 367 | 342 | 310 | 270 | 219 | 81 | 39 | 32 | 65 | 66 |
| Hoz de Jaca | 160 | 159 | 142 | 154 | 154 | 136 | 113 | 105 | 94 | 86 | 77 | 71 |
| El Pueyo de Jaca | 173 | 160 | 178 | 188 | 176 | 145 | 146 | 81 | 62 | 83 | 75 | 82 |
| Panticosa | 708 | 664 | 744 | 789 | 711 | 618 | 566 | 456 | 447 | 506 | 630 | 737 |
| Escarrilla | 221 | 213 | 202 | 196 | 183 | 169 | 162 | 146 | 133 | 132 | 170 | 252 |
| Lanuza | 208 | 205 | 183 | 191 | 183 | 165 | 157 | 141 | 0 | 0 | 0 | 43 |
| Sallent de Gállego | 691 | 733 | 677 | 636 | 578 | 877 | 554 | 776 | 641 | 607 | 763 | 1062 |
| Tramacastilla de Tena | 273 | 248 | 264 | 274 | 245 | 213 | 190 | 149 | 150 | 147 | 147 | 185 |
| Yésero | 288 | 311 | 293 | 254 | 248 | 201 | 194 | 141 | 61 | 53 | 81 | 71 |
| Bielsa | 959 | 1011 | 1253 | 1292 | 1113 | 919 | 748 | 654 | 475 | 439 | 456 | 511 |
| Bergua-Basarán | 619 | 638 | 606 | 508 | 443 | 402 | 215 | 15 | 2 | 13 | 40 | 47 |
| Broto | 361 | 368 | 373 | 377 | 308 | 349 | 301 | 301 | 243 | 204 | 237 | 254 |
| Oto | 308 | 322 | 307 | 291 | 270 | 239 | 171 | 126 | 108 | 92 | 97 | 79 |
| Sarvisé | 458 | 487 | 451 | 483 | 446 | 439 | 396 | 237 | 156 | 159 | 165 | 162 |
| Fanlo | 1023 | 1043 | 988 | 830 | 691 | 587 | 508 | 157 | 62 | 50 | 170 | 138 |
| Puértolas | 962 | 1013 | 1021 | 998 | 919 | 835 | 741 | 323 | 205 | 205 | 214 | 241 |
| Sin-Salinas | 323 | 357 | 457 | 468 | 355 | 250 | 195 | 122 | 100 | 94 | 83 | 82 |
| Tella | 273 | 286 | 324 | 486 | 439 | 488 | 422 | 329 | 322 | 227 | 198 | 184 |
| Linás de Broto | 380 | 395 | 370 | 373 | 385 | 351 | 233 | 142 | 84 | 72 | 60 | 59 |
| Torla | 586 | 607 | 510 | 519 | 538 | 463 | 379 | 302 | 285 | 291 | 287 | 269 |
|  |  |  |  |  |  |  |  |  |  |  |  |  |
| Zaragoza | 99118 | 111704 | 141350 | 173987 | 238601 | 264256 | 326316 | 479845 | 590750 | 622371 | 614905 | 674725 |
